# NAD + metabolism and function in innate and adaptive immune cells

**DOI:** 10.1186/s12950-025-00457-7

**Published:** 2025-08-01

**Authors:** Rebecca Mann, Victoria Stavrou, Sarah Dimeloe

**Affiliations:** Department of Immunology and Immunotherapy, School of Infection, Inflammation and Immunology, College of Medicine and Health, University of Birmingham, Birmingham, B15 2TT UK

## Abstract

Nicotinamide adenine dinucleotide (NAD+) plays a central role in cellular metabolism and energy production, supporting many biological processes. Recent studies highlight the significance of NAD + in regulation of immune cell function, with implications for our understanding of immune homeostasis, inflammation, and disease. This review reports our current understanding on the role of NAD + in the immune system, specifically in macrophages and T cells, facilitating their metabolic reprogramming during differentiation and activation. It offers an overview of NAD + biosynthesis within these immune cells, describes its role in the modulation of immune cell metabolism and effector function, and highlights potential therapeutic applications of NAD + modulation in immunological disorders including autoimmune diseases and cancer.

## Introduction

Nicotinamide adenine dinucleotide (NAD+) is an essential redox cofactor with a role in cellular processes including adenosine triphosphate (ATP) production, epigenetic regulation and DNA repair. NAD exists in both an oxidised and reduced form (NAD + and NADH respectively), and facilitates important metabolic pathways such as glycolysis, the tricarboxylic acid (TCA) cycle and oxidative phosphorylation (OXPHOS) by accepting and donating electrons at different pathway steps. NAD + can also be phosphorylated by NAD kinase (NADK1/2) to NADP+, which again has redox potential, and plays an important role in cellular reactive oxygen species (ROS) homeostasis among other activities. NAD + also acts as a co-substrate for many enzymes, through which NAD + is broken down into nicotinamide (NAM) and ADP-ribose. Key NAD-consuming enzymes include poly (ADP-ribose) polymerases (PARPs), which, for example, bind to and flag sites of DNA damage to initiate repair mechanisms; sirtuins, a family of deacetylases which exert activities including epigenetic modifications [1], and CD38, a multi-faceted glycohydrolase [2].

**Fig. 1:**
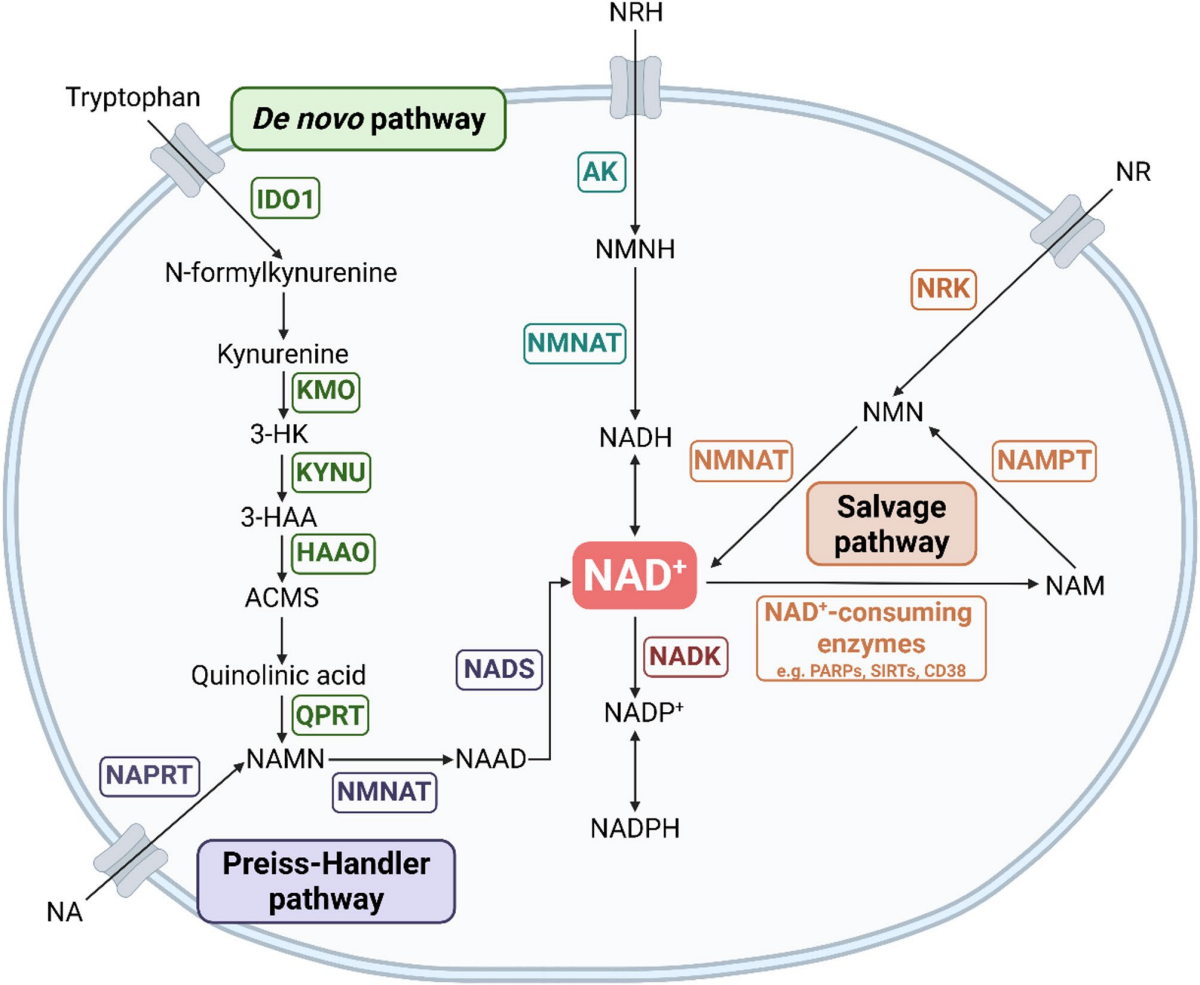
Overview of NAD + Synthesis Pathways. Schematic overview of the major NAD+ biosynthesis pathways, identifying key intermediate metabolites and enzymes (gene names in boxes)

NAD + is synthesised by three main pathways (Fig. 1). The *de novo* pathway generates NAD + from the essential amino acid tryptophan, a pathway rate-limited by the enzyme indoleamine 2,3-dioxygenase 1 (IDO1) [3], which is the first step in the pathway to convert tryptophan to kynurenine. Nicotinic acid (NA), also supplied from the diet, can enter the latter part of the *de novo* pathway via the Preiss-Handler pathway. NAD + can also be “recycled” after consumption by NAD-consuming enzymes via the salvage pathway, whereby NAM is converted to nicotinamide mononucleotide (NMN) via NAM phosphoribosyl-transferase (NAMPT) and back into NAD + via NMN adenylate transferases (NMNAT1-3) [4]. In addition, NAD + can be synthesised via nicotinamide riboside kinases (NRK1 and 2) which phosphorylate nicotinamide riboside (NR) into NMN, directing it into the salvage pathway [5, 6]. Yang et al.. also recently showed that NRH (reduced NR) can be converted into NAD + independently of the salvage pathway through activity of adenosine kinase (AK) and NMNAT [7].

Current literature describing NAD + biology in immune cells is primarily focused on macrophages and T cells, which are key players within the innate and adaptive immune systems respectively. Therefore, this review will summarise findings on these cell types, however, to fully understand the complex role of NAD + within the entire immune environment, further work is required on other immune cells such as dendritic cells and B cells, but also on immune cells subsets like regulatory T cells (TRegs) and unconventional populations. Upon activation, immune cells “metabolically reprogram” to support their energetic and synthetic demands. Macrophages exposed to pro-inflammatory stimuli, for example the bacterial component lipopolysaccharide (LPS) and cytokine interferon-gamma (IFN-γ) can polarise towards an “M1-like” phenotype. These respond rapidly to foreign pathogens, upregulating effector functions such as production of pro-inflammatory cytokines. They also increase glycolytic activity to support these energy-demanding functions. On the other hand, “M2-like” macrophages, which are induced by anti-inflammatory cytokines such as interleukin (IL)−4 and IL-13, resolve inflammation and repair tissue, and rely more heavily on OXPHOS for ATP generation [8]. However, whilst this paradigm is helpful to understand how distinct macrophage functions are underpinned by specific metabolic profiles, it is increasingly understood that macrophages rather exist across a spectrum of inflammatory and metabolic phenotypes [9]. In T lymphocytes, metabolic reprogramming is also described upon T cell receptor (TCR) cell ligation via cognate antigen alongside appropriate co-stimulation. T cells also upregulate glycolysis to support clonal expansion and acquisition of effector functions. They additionally increase glucose and glutamine oxidation in the TCA, driving increased OXPHOS [10–12].

As well as protection against pathogens, immune cells play vast and complex physiological roles, influencing autoimmunity and cancer to name just a few. Decreases in overall NAD + levels have been observed with increasing age [13] and in certain inflammatory diseases such as rheumatoid arthritis [14]. NAD + metabolism has also been explored in cancer, with clinical trials targeting NAD + synthesis pathways ongoing. As such, understanding the mechanisms by which NAD + impacts immune cell function is of great and timely importance. In this review, we will summarise current understanding of the role of NAD + and regulation of its synthesis in macrophage and T cell function and identify areas in this field requiring further clarification, as well as potential avenues for understanding the therapeutic potential of NAD + in immune-mediated disease.

### NAD + abundance in macrophages

Across different experimental models, with exposure to pro-inflammatory stimuli or with ageing, total intracellular NAD + in macrophages decreases [15–17]. This decrease is likely a consequence of increased NAD + consumption relative to NAD + synthesis that occurs upon M1-like polarisation of macrophages. LPS treatment significantly increases expression and NADase activity of CD38 in monocytes, and despite surface levels decreasing upon differentiation to macrophages, CD38 remains a highly expressed and active NADase in M1-like and aged macrophages [17–19]. LPS-stimulated bone marrow-derived macrophages (BMDMs) from *Cd38*-deficient mice were found to exhibit significantly higher NAD + abundance than wild-type controls, and similar levels of NADase activity as unstimulated or M2-like macrophages, implying NADase activity in LPS-stimulated BMDMs is near completely CD38 driven [17]. However, importance of PARPs in macrophage NAD + depletion has also been demonstrated. For example, in murine BMDMs, treatment with LPS and IFN-γ induced high amounts of mitochondrial ROS, which induced DNA damage and PARP activity, consuming NAD+ [16].

### NAD + biosynthesis in macrophages

In activated macrophages, NAD + is reported to be predominantly synthesised via NAD + salvage, and NAMPT is shown to play a role in macrophage polarisation and metabolic reprogramming. Across murine and human macrophage models, M1-like macrophage polarisation coincides with increased NAMPT expression [16, 20, 21] and several reports have demonstrated that NAMPT activity is important for sustaining sufficient NAD + levels to maintain the high levels of glycolysis required for M1-like polarisation and function. This is partly through supporting activity of the NAD-dependent glycolytic enzyme, glyceraldehyde-3-phosphate dehydrogenase (GAPDH), facilitating increased production of pro-inflammatory cytokines including tumour necrosis factor-alpha (TNF-α) and IL-6 [16, 20, 22]. In agreement, Chini et al.. showed that supplementation with NRH boosted NAD + levels in both murine BMDMs and THP1 cell line-derived macrophages, promoting polarisation towards an M1-phenotype and increasing expression of a pro-inflammatory profile of genes [23]. This was also demonstrated in a disease context where, in mice with liver ischemia–reperfusion injury, a condition driven by inflammation, FK866 (a specific and potent NAMPT inhibitor) decreased intracellular NAD + concentrations in liver-resident macrophages and reduced abundance of M1-like macrophages (defined by expression of inducible nitric oxide synthase (iNOS) and TNF-α), which improved symptoms and survival [24].

Macrophage NAMPT expression within the tumour microenvironment has been associated with cancer, but with differing conclusions. Mice with a myeloid cell-specific knockout of NRE1 (an element in the first intron of *Nampt*) were reported to exhibit increased tumour growth in a melanoma model [20]. However, a recent report showed that in a murine colorectal cancer model, a myeloid cell-specific knockout of *Nampt* resulted in decreased tumour mass, associated with reduced abundance of M2-like tumour-associated macrophages (TAMs), a cell subset generally considered to be pro-tumourogenic. This was attributed to the fact that in a lactate-rich environment (such as the tumour microenvironment), NAMPT-dependent replenishment of NAD + maintains oxidation of lactate to pyruvate, which promoted stabilisation of the transcription factor, hypoxia-inducible factor-1 alpha (HIF-1α), contributing to differentiation of an M2-like immunosuppressive macrophage phenotype [25]. Thus, overall effects of NAD + abundance and modulation within macrophages in cancer may also depend upon specific microenvironmental factors of distinct tumour types.

### The *de novo* NAD + synthesis pathway in macrophages

The *de novo* pathway has been studied extensively in the context of cancer, as IDO1 is highly expressed by tumour cells across various types of cancer [26]. This creates an increased ratio of kynurenine to tryptophan, which is proposed to contribute to an immunosuppressive tumour microenvironment, in part (but not entirely) through activity of kynurenine on the aryl hydrocarbon receptor (AhR) [27, 28]. In oral squamous cell carcinoma, increased tumour infiltration of macrophages expressing high levels of IDO1 was also associated with worse prognosis [29].

In human monocyte derived macrophages (MDMs), Minhas et al.. reported no change in NAMPT abundance upon LPS stimulation, however a distinct pattern of *de novo* pathway enzyme expression arose, whereby enzymes early in the pathway (IDO1 and kynureninase (KYNU)) were upregulated upon activation but the further downstream quinolinate phosphoribosyltransferase (QPRT) decreased (Fig. 1). This was shown to create a “bottleneck” effect and despite sufficient precursors, flux through to NAD + production was limited. Tracing deuterium-labelled kynurenine revealed that at rest, around 40% of total NAD + was synthesised by the *de novo* pathway, therefore this change in enzyme expression could conceivably contribute to macrophage NAD + depletion upon LPS stimulation [15]. This pattern of enzyme expression in M1-like macrophages was also demonstrated across various models of in vitro human MDM stimulation [30].

### NAD + depletion and macrophage inflammatory function

Whilst some studies highlight the importance of NAMPT in M1-like polarisation and function, other reports have shown that depletion of NAD^+^ itself contributes to an M1-like phenotype. Peritoneal exudate macrophages (PEMs) from both *Ido1* and *Qprt*-deficient mice exhibited reduced total NAD + compared to wild-type, and mimicked LPS-stimulated PEMs, with decreased OXPHOS and increased glycolysis [15]. *Qprt*-deficient murine macrophages and human MDMs treated with a pharmacological inhibitior of QPRT demonstrated increased expression of M1-associated surface markers and increased production of inflammatory cytokines e.g. IFN-γ and IL-1β [15]. In agreement, Liu et al.. showed that across several macrophage models, NAD + supplementation with NMN alongside LPS suppressed production of IL-6 and IL-1β. NMN reduced COX-2 expression in LPS-treated PEMs, which is involved in the synthesis of the pro-inflammatory mediator prostaglandin E_2_, possibly explaining these anti-inflammatory effects [31].

Similarly, Wang et al.. found that upregulation of CD38 expression upon LPS stimulation in both murine BMDMs and human MDMs is regulated by the histone modifier pax transactivation domain-interacting protein (PTIP) and interestingly, this was shown to be a crucial component of M1-like polarisation. Knockout of *Ptip* reduced *Cd38* expression, which increased total intracellular NAD+. This was associated with reduced glycolysis and increased OXPHOS, reflecting a blunting of characteristic metabolic reprogramming in LPS-stimulated BMDMs. PTIP deficiency was also associated with reduced M1-associated surface markers and cytokine expression [32].

NAD + depletion is also reported to impact macrophage inflammatory signalling pathways, of which dysregulation contributes to inflammatory disease. The NOD-like receptor protein 3 (NLRP3) inflammasome is a protein complex which mediates caspase 1 activation, initiating macrophage inflammatory cascades [33]. It was recently reported that in unstimulated BMDMs, treatment with known NLRP3 inflammasome ‘second signals’, ATP or nigericin alone, did not induce activation of caspase-1, but alongside FK866 treatment did result in caspase-1 cleavage, indicating that NAD + depletion can act as a priming signal for NLRP3 inflammasome activation [34]. Likewise, NLR family caspase recruitment domain containing 5 (NLRC5), a NOD-like receptor which senses intracellular pathogen and damage associated molecular patterns [35], was shown to upregulate in murine BMDMs in response to NAD + depletion and thereby interact with other inflammatory cell mediators such as NLRP3 and NLRP12 to coordinate lytic cell death pathways, propagating pathological inflammation. In murine models of inflammatory diseases such as colitis, *Nlrc5*-deficient mice showed improved survival and reduced inflammatory-mediated tissue damage [36]. Mycobacterium tuberculosis (Mtb), which infects and replicates within macrophages, has also been demonstrated to exploit this mechanism. Mtb produces tuberculosis necrotizing toxin (TNT), an NAD+-depleting glycohydrolase and in THP1-derived macrophages, mTB-derived TNT depleted intracellular NAD^+^, which was shown to trigger necroptosis, a type of programmed cell death advantageous for mTB dispersal and survival [37].

### NAD + regulation in T cells

Similar to macrophages, the salvage pathway has been described as the primary source of NAD + in T cells. Using CRISPR-Cas9-based genetic screens in T cell receptor (TCR)-stimulated Jurkat T cell line, Wang et al.. demonstrated that NAMPT plays a critical role in T cell NAD + synthesis. NAMPT expression is induced upon T cell activation, a process controlled by the transcription factor Tubby (TUB) which senses TCR stimulation, increasing intracellular NAD + levels [38]. In contrast to macrophages, overall T cell NAD + levels increase in response to activation, perhaps indicating a greater ratio of NAD + synthesis to consumption than in macrophages. Through high levels of NAD + production, the salvage pathway appears to act as a conduit for naive T cells to exit their quiescent state and enter into the cell cycle to support clonal expansion. Increasing TCR affinity for antigen is associated with higher levels of cellular NAD+, which contributes to the rate of cell cycle entry through NAD-dependent GAPDH activity and downstream central carbon metabolism. At the single-cell level, NADH concentrations can predict division potential of both T cells (CD4^+^ and CD8^+^) and B cells before their initial division [39], suggesting NADH levels could even serve as a biomarker for assessing proliferative heterogeneity within T cell populations.

Of note, NAD + is also implicated in regulating T cell population size by promoting apoptosis, albeit through extracellular pathways. Specifically, NAD + released during tissue injury and inflammation has been described to activate the P2RX7 purinergic receptor (P2X7) on mature T cells, initiating a sequence of steps which ultimately induce cell death via apoptosis. This is known as NAD-induced cell death, a process characterised by increased calcium flux, shedding of CD62L and depolarisation of mitochondrial membrane potential [40]. The apparent inconsistency with findings described above may relate to this activity being exerted extracellularly via cell surface receptor recognition.

### NAD + and T cell differentiation

Alongside clonal expansion, activated T cells also differentiate into effector populations with distinct inflammatory functions during immune responses. NAD + metabolism has been identified as a key regulator of T cell differentiation in various settings. For example, in a murine experimental autoimmune encephalomyelitis (EAE) model, direct administration of NAD + was shown to regulate CD4^+^ T cell differentiation independently of conventional transcription factors, but via tryptophan hydroxylase-1, an enzyme involved in serotonin biosynthesis. In this way, NAD + supplementation converted T helper 1 (Th1)-like, pro-inflammatory IFN-γ producing cells into cells also producing the immunosuppressive cytokine IL-10 [41]. Conversely, in a murine sepsis model, NR provision effectively increased NAD + levels in T cells, which expanded Th1 and T helper 2 (Th2) subsets and reduced TReg frequency. NR treatment also alleviated sepsis-driven T cell exhaustion, decreasing programmed cell death protein 1 (PD-1) expression in CD4^+^ T cells. Consistently, T cell proliferative capacity and survival were enhanced, associated with decreased bacterial load, reduced organ damage and mortality [42]. In another study, NR supplementation was conversely found to blunt pro-inflammatory CD4^+^ Th1 and T helper 17 (Th17) activity in healthy volunteers and patients with psoriasis. Mechanistically, this was linked with increased NAD + levels, enhanced arginine and fumarate biosynthesis via arginosuccinate lyase activity and activation of the transcription factor Nuclear Factor Erythroid 2 Related Factor 2 (NRF2). This initiated an anti-oxidant response, decreasing ROS levels, Th17 polarisation and IL-17 production [43]. Of note, by modulating NAD + levels, CD38 has also been described to regulate T cell differentiation, activation, development, and characteristics of T cell health, which has been summarized in greater depth in another review [44]. Taken together, these studies highlight potential for NAD + supplementation to impact T cell differentiation during infection and inflammatory disease, but indicate potentially distinct effects of precursor versus direct NAD + administration, and identify capacity for increased NAD + levels to impact diverse aspects of T cell signalling and metabolism, in agreement with its broad and diverse cellular roles.

### NAD + and T cell antitumor responses

Tumour infiltrating lymphocytes (TILs) often demonstrate functional impairment accompanied by metabolic alterations. In relation to NAD+, TILs exhibit impaired TCR-dependent TUB activity and NAMPT upregulation, leading to decreased NAD + levels. This is associated with impaired glycolysis, mitochondrial dysfunction, and decreased ATP production, indicating decreased NAD + levels could contribute to decreased TIL function in tumour microenvironments. Of note, in this study NAM supplementation restored T cell NAD+, rescuing metabolic activity, enhancing tumour-killing capacity and extending survival time. Remarkably, supplementation also improved efficacy of both chimeric antigen receptor T-cell (CAR-T) therapy and anti-PD-1 immune checkpoint blockade in murine models [38]. In another study, enhancing NAD + levels via NAM provision was also shown limit exhaustion of both CD4^+^ and CD8^+^ T cells in vitro and promote differentiation of effector memory and terminal effector T cells. Specifically, NAM limited upregulation of inhibitory receptors including CD39 and TIM-3, and restored IL-2 and TNF-α expression. This was correlated with decreased mitochondrial ROS and expression of the exhaustion-associated transcription factor, thymocyte selection-associated high mobility group box (TOX) [45], consistent with previous studies linking ROS with TOX upregulation [45, 46]. Yu et al. also demonstrate that CD8^+^ TILs in a murine melanoma model demonstrate features of exhaustion, including increased PD-1 signalling, linked to mitochondrial depolarisation. Here, administration of NR restored TIL mitochondrial fitness, increased anti-tumour function and improved sensitivity to anti-PD-1 treatment [46]. Collectively these studies show that boosting NAD + synthesis can prevent or reverse T cell exhaustion, highlighting potential for modulating T cell NAD + metabolism to improve outcomes with immune-directed therapies in cancer.

Related to this, boosting intracellular NAD + levels in a murine melanoma model was shown in tumor specific T cells to prevent induction of senescence, a state of permanent growth arrest, associated altered functional capacity and a senescence-associated secretory phenotype (SASP). In this study, NAD + supplementation with NMN enhanced survival and decreased tumour size. This was associated with T cell SASP suppression (including of pro-inflammatory cytokines), enhanced mitophagy, and restored T cell frequencies [47].

Although the salvage pathway is the primary focus in many studies, the *de novo* NAD + synthesis pathway has also been shown to regulate CD8^+^ T cell metabolic and functional state, relevant to anti-tumour immunity. Specifically, via genetic manipulation of *Kynu* in T cells, it was shown that NAD + derived via this pathway supports CD8^+^ T cell glycolysis, OXPHOS, and effector functions such as cytokine secretion and cytotoxicity. Mechanistically, this was shown to involve NAD+-dependent acetylation and degradation of phosphatase and tensin homolog deleted on chromosome 10 (PTEN), an important negative regulator of signalling via PI3K/mTOR, which promotes T cell metabolic reprogramming [48].

### NAD + in autoimmune diseases

Imbalanced T cell NAD + homeostasis has also been described in autoimmune disease, with manipulation of NAD + abundance also demonstrating therapeutic potential [41, 43, 49, 50]. For example, direct provision of NAD + in vivo was shown to inhibit and reverse EAE progression, promoting myelin and axonal regeneration, associated with suppressed T cell differentiation [41]. Conversely, Bruzzone et al. reported that depleting NAD + levels with FK866 also reduced clinical symptoms in EAE and demyelination. Here, FK866 appeared specifically targeted activated T cells, impairing their proliferation and reducing production of IFN-γ and TNF-α [49].

Patients with immune thrombocytopenia (ITP) exhibit decreased frequency and function of TRegs, a specialized immune population that plays a vital role in preventing autoimmune diseases. Decreased plasma NAD + levels and overall CD4^+^ T cell frequencies are also reported. In an ITP murine model, NAM provision was shown to increase TReg frequency, ameliorating thrombocytopenia. Similarly, in vitro, NAM provision to patient samples restored cytosolic NAD^+^ level in the CD4^+^ T cells, promoting TReg differentiation. Mechanistically, NAM was found to promote acetylation and stability of the hallmark TReg transcription factor Foxp3 via Sirt1 inhibition [50].

## Conclusion

This review highlights the significance of NAD + within the immune cell function and demonstrates that numerous cellular functions are controlled by NAD+, as illustrated by manipulation of key biosynthesis pathways or NAD-consuming enzymes. Interestingly in macrophages, NAD + abundance appears to decrease upon pro-inflammatory M1-like polarisation, especially during inflammation and ageing and may even play a role in promoting this, while in T cells, NAD + levels increase upon activation, proportionate to TCR signal strength and differentiation state. These observations highlight cellular NAD + status is a key control point for immune cell differentiation and moreover that distinct immune cells exhibit distinct NAD + requirements to sustain homeostasis. Manipulation of cellular NAD + levels demonstrates clear potential to influence inflammatory and antitumour responses with implications for therapeutic modulation in many diseases. Indeed, several clinical trials are already underway to interrogate the potential for NAD + precursors like NMN and NR in disease contexts including neurodegenerative disorders and cancer [51–53]. Although these are safe and well tolerated, their impact on immune response is yet to be explored in detail and will require future focus, particularly since NAD + synthesis pathways and cellular status may have distinct capacity to influence differentiation and activity of innate and adaptive immune cells. Key open questions around NAD + biology in immune cells include how NAD + synthesis, abundance and activity evolve during the differentiation of populations into effector, memory and ultimately exhausted states and implications of this for cellular metabolism and function. In addition, recent studies have highlighted that dynamic changes in subcellular localisation of NAD + synthesis impact cell differentiation trajectories, which remains to be explored in immune cells. Finally, another key area to explore relates to whether organism-level decline in NAD + abundance with ageing also occurs at the level of individual immune cells and what this means for immune function as we age.

**Table:** 

	Macrophages	T cells
Major NAD + synthesis pathway	NAMPT/NAD + salvage pathway. Role of *de novo* synthesis pathway also described [15, 16]	NAMPT/NAD + salvage pathway. Role of *de novo* synthesis pathway also described [39, 48]
Change in NAD + abundance upon stimulation	Decrease upon LPS stimulation [15, 16]	Increase upon CD3/28 stimulation [39]
Effect of NAMPT/NAD + salvage pathway inhibition	Reduced M1-like surface markers, inflammatory cytokine production, glycolysis and OXPHOS in LPS stimulated murine macrophages [16]. Reduced abundance of M1-like macrophages and alleviated symptoms in murine model of liver ischaemia-reperfusion injury [24]. Induced caspase 1 cleavage alongside known NLRP3 inflammasome second signals in BMDMs [34].	Impaired T cell activation and cell cycle entry. Decreased IFN-γ and TNF-α production. Reduced clinical symptoms in experimental autoimmune encephalomyelitis and demyelination [39, 49].
Effect of disruption of *de novo* NAD + synthesis pathway	IDO1 and QPRT deficiency decreased OXPHOS and increased glycolysis. QPRT deficiency increased expression of M1-like surface markers and pro-inflammatory cytokine production [15].	KYNU deficiency decreased glycolysis and OXPHOS, decreased IFN-γ expression [48].
Effect of loss of CD38 function	Blunted characteristic LPS-induced metabolic reprogramming in BMDMs. Reduced M1-associated surface markers and cytokine production [32].	Varied effects including both promotion and suppression of activation. Roles for both NAD + depletion and cyclic ADP-ribose generation [44].
Effect of augmenting NAD + via precursor provision	NMN provision suppressed inflammatory cytokine production and reduced COX-2 expression in LPS-treated PEMs [31] NRH provision promoted expression of pro-inflammatory gene profile in BMDMs and THP1-derived macrophages [23]	NR provision relieved sepsis-driven T cell exhaustion [42] and blunted pro-inflammatory CD4^+^ Th1 and Th17 activity in healthy volunteers and patients with psoriasis [43]. NAM provision enhanced efficacy of CAR-T therapy and anti-PD-1 immune checkpoint blockade in murine cancer models [38], decreased T cell exhaustion in vitro [45] and increased TReg frequency, ameliorating autoimmune thrombocytopenia in a murine model [50]

## Data Availability

No datasets were generated or analysed during the current study.

## Abbreviations

ACMS: Aminocarboxymuconic semialdehyde
AhR: Aryl hydrocarbon receptor
AK: Adenosine kinase
ATP: Adenosine triphosphate
BMDMs: Bone marrow derived macrophages
CAR-T: Chimeric antigen receptor T-cell
EAE: Experimental Autoimmune Encephalomyelitis
GAPDH: Glyceraldehyde-3-phosphate dehydrogenase
HAOO: 3-Hydroxyanthranilic acid 3,4 dioxygenase
HIF-1α: Hypoxia-Inducible Factor 1-alpha
IDO1: Indoleamine 2,3-dioxygenase 1
IFN-γ: Interferon-gamma
IL: Interleukin
iNOS: Inducible nitric oxide synthase
IRs: Inhibitory receptors
ITP: Immune thrombocytopenia
KMO: Kynurenine 3-monooxygenase
KYNU: Kynureninase
LPS: Lipopolysaccharide
MDMs: Monocyte derived macrophages
Mtb: Mycobacterium tuberculosis
NA: Nicotinic acid
NAAD: Nicotinic acid adenine dinucleotide
NAD: Nicotinamide adenine dinucleotide
NADH: Reduced form of NAD
NADP: NAD phosphate
NADPH: Reduced form of NADH
NADS: NAD synthetase
NADK 1/2: NAD kinase 1/2
NAM: Nicotinamide
NAMN: Nicotinic acid mononucleotide
NAMNAT: Nicotinamide mononucleotide adenylyltransferase
NAMPT: NAM phosphoribosyl-transferase
NAPRT: Nicotinic acid phosphoribosyl transferase
NCID: NAD induced cell death
NLRC5: NLR caspase recruitment domain (CARD) containing 5
NLRP3: NLR family pyrin domain-containing 3
NLRP12: NLR Family Pyrin Domain Containing 12
NMN: Nicotinamide mononucleotide
NMNAT: Nicotinamide mononucleotide adenylyltransferase
NMNH: Reduced form of NMN
NRK: Nicotinamide riboside kinase
NR: Nicotinamide riboside
NRH: Reduced NR
NRF2: Nuclear Factor Erythroid 2 Related Factor 2
OXPHOS: Oxidative phosphorylation
PARPs: Poly(ADP-ribose) polymerase
P2X7: P2RX7 purinergic receptor
PARPs: Poly (ADP-ribose) polymerase
PD-1: Programmed cell death protein 1
PEMs: Peritoneal exudate macrophages
PTEN: Phosphatase and tensin homolog deleted on chromosome 10
PTIP: Pax transactivation domain-interacting protein
QPRT: Quinolinate phosphoribosyl transferase
ROS: Reactive oxidative species
SASP: Senescence-associated secretory phenotype
SIRTs: Sirtuins
TAMs: Tumour-Associated Macrophages
TCA: Tricarboxylic acid cycle
TCR: T cell receptor
Th 1: helper 1
Th17: T helper 17
Th2: T helper 2
TILs: Tumour infiltrated lymphocytes
TNF-α: Tumour necrosis factor-alpha
TNT: Tuberculosis necrotizing toxin
TOX: Thymocyte selection-associated high mobility group box
Treg: Regulatory T cells
TUB: Tubby
3-HK: 3-Hydroxykynurenine
3-HAA: 3-Hydroxyanthranilic acid
